# The Digital Revolution in Cardiac Ischemia: Artificial Intelligence (AI)-Enhanced Detection, Diagnosis, and Risk Stratification

**DOI:** 10.7759/cureus.95059

**Published:** 2025-10-21

**Authors:** Ahmed Khalifa, Mostafa Abdulaziz, Syed Shahzil Parvez, Ahmed Salem, Hafsa S Panhwer, Usman Saleem, Mahmoud Eldeeb, Tariq Weld-Ali, Maryam Mosaad

**Affiliations:** 1 Cardiology, Frimley Park Hospital, Frimley Health NHS Foundation Trust, Frimley, GBR; 2 Cardiology, University Hospital Monklands, Airdrie, GBR; 3 General Medicine, Frimley Health NHS Foundation Trust, Frimley, GBR; 4 Internal Medicine, The Grange University Hospital, Cwmbran, GBR; 5 Internal Medicine, Frimley Park Hospital, Frimley Health NHS Foundation Trust, Frimley, GBR; 6 Accident and Emergency Medicine, Royal Surrey County Hospital, Royal Surrey NHS Foundation Trust, Guildford, GBR; 7 General Practice, The Cambridge Practice, Aldershot, GBR; 8 Emergency, Sharq El Madina Hospital, Egyptian Ministry of Health and Population, Alexandria, EGY

**Keywords:** artificial intelligence, cardiac ischemia, deep learning, digital health, electrocardiogram, machine learning, myocardial infarction, wearable devices

## Abstract

The digital revolution in cardiac ischemia has changed with ongoing applications of artificial intelligence (AI) to overcome the limitations of traditional diagnostic tools and risk scores. Recent advances in machine learning and deep learning (DL) have enabled convolutional neural networks to detect myocardial infarction with high accuracy, including subtle non-ST-elevation occlusion events that often elude human readers. AI-driven analyses of coronary CT angiography now automate plaque quantification and stenosis assessment, while DL-based fractional flow reserve computation reduces evaluation time without compromising diagnostic performance. In cardiac MRI and perfusion imaging, AI algorithms perform real-time myocardium segmentation and ischemia detection at expert levels. Wearable device integration offers continuous out-of-hospital monitoring for the early detection of ischemic events. Despite challenges related to algorithmic bias, clinical workflow integration, and validation across diverse populations, current evidence demonstrates that AI-enhanced tools not only match but often surpass traditional methods and expert interpretation. As multimodal AI integration, personalized risk prediction models, and advanced wearable technologies continue to evolve, AI promises to transform cardiac ischemia management by enabling earlier detection, more accurate diagnosis, and refined risk stratification, ultimately improving patient outcomes.

## Introduction and background

Cardiovascular disease remains the leading cause of mortality worldwide, with ischemic heart disease accounting for a large proportion of these deaths [1,2]. Myocardial ischemia due to coronary artery disease can present acutely or chronically, and its early detection, coupled with appropriate risk stratification, is essential for optimizing revascularization and preventive treatment [3-5].

Conventional diagnostic tools and risk scores have limitations in sensitivity and precision. The 12-lead electrocardiogram (ECG) and cardiac troponin biomarkers form the cornerstone of acute myocardial infarction (MI) diagnosis. Yet, subtle ischemic ECG changes (e.g., in occlusion MI, OMI, without ST-elevations) are often missed by human readers [6-9].

Coronary CT angiography, myocardial perfusion imaging, or stress echocardiography provides valuable anatomic and functional information, but they require significant expertise to interpret and may not be widely accessible [10,11]. Traditional risk models, such as the Framingham Risk Score, use only a handful of clinical factors and have modest accuracy in individual patients [12-14].

In recent years, artificial intelligence (AI) techniques have shown potential to address several limitations in cardiovascular medicine. Machine learning (ML) can discover complex patterns in large datasets, while deep learning (DL) uses multilayer neural networks to automatically extract nuanced features from raw waveforms or images [15,16]. These approaches have already enabled "augmented" ECG interpretation that rivals expert cardiologists [15], automated analysis of imaging that matches invasive gold standards [17], and predictive models that outperform conventional risk scores [18]. Given these successes, there is a growing digital revolution in how we detect and manage cardiac ischemia using AI. This narrative review provides a comprehensive overview of current applications of AI in the context of cardiac ischemia, focusing on work from 2018 to 2025.

## Review

Methods

This review was designed as a narrative synthesis of recent, clinically relevant applications of AI to cardiac ischemia. The scope covered electrocardiogram (ECG) signal analysis, coronary imaging, wearable and remote monitoring devices, and multimodal prognostic modeling, emphasizing translational evidence, diagnostic performance, workflow impact, and prognostic value.

We searched the Medical Literature Analysis and Retrieval System Online via PubMed, Embase, Scopus, Web of Science, the Institute of Electrical and Electronics Engineers Xplore digital library, and the Cochrane Library (from 2018 to September 19, 2025). We screened arXiv and major cardiovascular society websites: the European Society of Cardiology, the American College of Cardiology, the American Heart Association, and the Society of Cardiovascular Computed Tomography. Search queries combined AI terms (such as “machine learning” and “deep learning”) with modality and disease keywords, for example, electrocardiogram, coronary computed tomographic angiography, late gadolinium enhancement (LGE), ischemia, MI, and coronary artery disease, as well as diagnostic and prognostic terms. Reference lists of eligible and highly cited works were snowballed to capture influential studies.

We prioritized human studies in adults with explicit AI components relevant to ischemia and coronary artery disease end points (for example, diagnostic accuracy, quantification of plaque and stenosis, ischemic burden, and risk prediction of major adverse cardiovascular events, MACE). Preference was given to peer-reviewed, multicenter, externally validated, prospective, or outcome-linked studies. Single-center or small retrospective training sets were included when methodologically influential or when they illustrated a clinically important theme. We excluded animal- or simulation-only work without human validation, as well as editorials or letters without new data. When cohorts overlapped, the most complete or the latest peer-reviewed report was favored. Findings were synthesized thematically by clinical task and modality, comparing AI performance with clinical baselines or conventional tools and highlighting externally validated, prospective, or outcome-linked evidence. No quantitative pooling or meta-analysis was attempted due to methodological diversity and the narrative intent.

AI-enhanced electrocardiogram analysis for ischemia detection

AI-Augmented ECG Interpretation

AI is reshaping noninvasive cardiac electrophysiology, especially ECG-based analyses, by enabling earlier recognition of ischemia-related signals and subtle precursors that manual review often overlooks [19,20].

AI can significantly enhance the diagnosis of cardiac ischemia from electrocardiograms (ECGs). DL models, trained on extensive ECG databases, are capable of detecting and locating patterns of MI with remarkable accuracy [21,22]. In particular, convolutional neural networks (CNNs) have proven highly effective for ECG analysis. A 2022 literature review by Xiong et al. surveyed 59 DL studies for MI detection/localization via ECG and found that CNN-based models achieved over 97% accuracy on internal validation datasets [21]. These models can identify subtle changes in the ECG signal that may be imperceptible to clinicians.

AI algorithms are capable of detecting subtle and distributed perturbations in the ST and T wave segments across multiple ECG lead patterns that can signify the presence of an occlusion-related MI-even in cases where the hallmark finding of classic ST-segment elevation is absent [22-25].

Al-Zaiti et al. developed an ML model for precisely this challenge: detecting occlusion MI in patients without ST-elevations. Trained on over 7,000 patients’ ECGs, their model outperformed experienced physicians and commercial ECG software, substantially improving sensitivity for acute coronary occlusions. When combined with clinicians' judgment, the AI-driven OMI risk score enabled reclassification of roughly one in three chest-pain patients, ensuring many “hidden” OMIs would receive urgent reperfusion therapy [22].​​​​​

DL for MI Detection and Localization

Beyond binary detection of MI, AI is used to localize ischemic regions via ECG. CNNs and recurrent networks can be trained to recognize patterns corresponding to anterior, inferior, or posterior MI, etc., based on 12-lead ECG inputs. In general, algorithms using the full 12-lead information have outperformed those with fewer leads [21,26,27]. One notable deep-learning model, trained on resting 12-lead ECGs, identified regional left ventricular (LV) akinesis as a surrogate of prior (silent) MI, thereby flagging patients who might merit further evaluation [28].

AI-ECG tools have also been shown to detect other latent cardiovascular conditions (like LV dysfunction or arrhythmias) from normal-appearing ECGs [15,29]. These findings underscore the concept that high-dimensional ECG data contain rich latent information that AI can unlock, effectively turning the ECG into a powerful, noninvasive biomarker beyond human visual interpretation.

Importantly, a recent study investigated the use of an AI system in conjunction with smartwatch-based nine-lead ECGs for the diagnosis of acute coronary syndromes (ACS). The research, which compared the AI's performance on smartwatch ECGs to traditional 12-lead ECGs, found a high concordance and comparable diagnostic accuracy between the two methods. These findings suggest that an AI model trained on standard ECG data can be effectively applied to the asynchronous, multichannel images from a smartwatch, holding promise for aiding ACS diagnosis in various locations [30,31]. This suggests that consumer devices, when paired with robust AI analysis, could substantially improve early ischemia detection outside of hospital settings. Other reports have documented that even single-lead smartwatch ECGs can capture ischemic ST changes; for instance, there are case reports of ST-elevation MI visible on an Apple Watch ECG trace at symptom onset.

The accuracy of AI models in ECG-based ischemia detection generally surpasses traditional methods. Classic computer ECG interpretations rely on fixed criteria and have limited sensitivity, whereas AI models learn from waveform morphology in a data-driven way. For detecting acute MI, several DL algorithms have reported sensitivities and specificities in the 90%-99% range on test sets. For example, one deep CNN achieved >99% sensitivity for MI on a public dataset by learning subtle QRS and T-wave changes [21].

However, it is important to contextualize these performance metrics. Extremely high accuracies often reflect training and testing on idealized or retrospective data. In prospective clinical settings with noisy data, performance may be lower. Indeed, when Al-Zaiti et al.’s 2023 OMI model was externally validated, its area under the curve (AUC) was about 0.89, and it still missed some occlusions [22]. Nonetheless, convergent evidence from head-to-head comparisons shows that data-driven AI approaches not only surpass classic, fixed-threshold ECG rules but also rival, and in many settings outperform, the diagnostic judgments of expert cardiologists [16,22,23,32,33].

A key advantage of modern DL models is their ability to leverage ancillary data alongside ECG signals for enhanced diagnostic accuracy. Studies have shown that models that combine ECG findings with clinical information such as age and sex can improve their ability to detect MI in the emergency setting. This multimodal approach allows AI to integrate a broader range of clinical information, leading to more robust risk stratification and patient triage [22,34].

Multitask networks are also emerging; for example, a 2025 study by Lin et al. introduced a multitask DL model (ECG-MACE) that simultaneously assessed ECGs for risk of four outcomes (MI, heart failure, stroke, and mortality) at one year, achieving an AUC of 0.85 for predicting MI and even outperforming clinical risk scores over longer timeframes [1].

While most AI in ECG involves signal-processing via DL, natural language processing (NLP) can play a supporting role in ECG-based care. For instance, NLP can be applied to physician ECG interpretation notes or cardiology consult reports to extract descriptors of ischemia, such as ST depression in anterior leads. These unstructured text data can then feed into ML models for diagnosis or risk stratification. However, there is limited literature on NLP improving ischemia detection. One area NLP has been tested in is capturing contextual risk factors from electronic health records (EHRs) that might affect post-MI outcomes. In a 2022 study, Brown et al. attempted to use NLP on clinical notes to identify social risk factors (e.g., lack of support, substance use) for patients hospitalized with acute MI to see if this improved 30-day readmission predictions. The addition of those NLP-extracted factors did not significantly enhance the ML model's performance [35]. This suggests that while NLP can automate data extraction from text, the selected factors in that study did not materially change risk stratification, or that more sophisticated NLP (capturing nuanced clinical narratives) may be needed.

**Figure 1:**
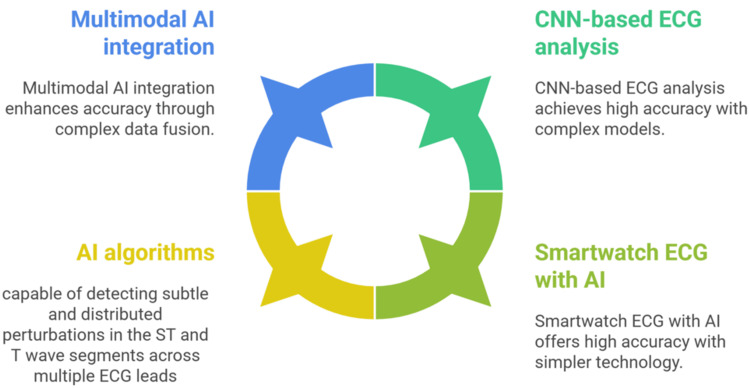
AI-enhanced electrocardiogram analysis for ischemia AI: artificial intelligence; CNN: convolutional neural network; ECG: electrocardiogram Image credit: The image was created by the authors Ahmed Khalifa and Mostafa Abdulaziz, showing features of AI-enhanced ECG analysis for ischemia Source: [19-36]

CNNs can interpret echocardiographic videos to detect wall motion abnormalities consistent with ischemia and even predict outcomes. One 2021 study showed that DL on echo videos improved mortality prediction compared to human assessments. Such an Echo AI could be considered part of the ischemia detection toolkit, especially where other imaging is not available. Additionally, nuclear imaging (single-photon emission CT, SPECT/positron-emission tomography) has benefitted from AI for noise reduction, resolution enhancement, and, as mentioned, direct risk prediction from scans (Figure 1) [1,36].

AI in cardiac imaging for ischemia

AI in CCTA

CCTA offers a noninvasive view of the coronary anatomy and atherosclerotic plaque. AI techniques, especially DL image segmentation, have dramatically improved the efficiency of CTA interpretation. AI can automatically identify the coronary artery lumen and quantify stenosis, reducing the need for tedious manual analysis. For example, an AI-based quantitative coronary angiography tool was developed using DL to delineate lumen borders on invasive angiograms, achieving 89% sensitivity in detecting lesions and closely matching human experts on measurements like diameter stenosis and lesion length [37].

The AI can automatically quantify plaque burden and stenosis severity on CTA images; its measurements showed strong agreement with invasive intravascular ultrasound (IVUS), and importantly, the AI analysis had prognostic value for future cardiac events [38].

In other words, patients flagged by the AI as having high-risk plaques on CTA were more likely to suffer MIs down the line, suggesting the model's output could be used for risk stratification. A key finding from a study on a DL system for coronary computed tomography angiography (CCTA) is that patients identified by the AI as having high-risk plaques were more likely to experience future MIs. This suggests the model's output could be effectively used for risk stratification in clinical practice. The study's authors created a system that automatically quantifies plaque burden and stenosis severity on CCTA images, with measurements showing strong agreement with invasive IVUS. The AI-powered tool has prognostic value for predicting future cardiac events, a finding that was externally validated across multiple centers. The study specifically demonstrated that a DL-based total plaque volume of 238.5 mm³ or higher was associated with an increased risk of MI [38].

AI has advanced CCTA by enabling the noninvasive estimation of fractional flow reserve (FFR). The introduction of DL-based FFR computation methods enhances the accuracy of stenosis assessment and substantially reduces operational time, making the technology more efficient for clinical use [39].

**Figure 2:**
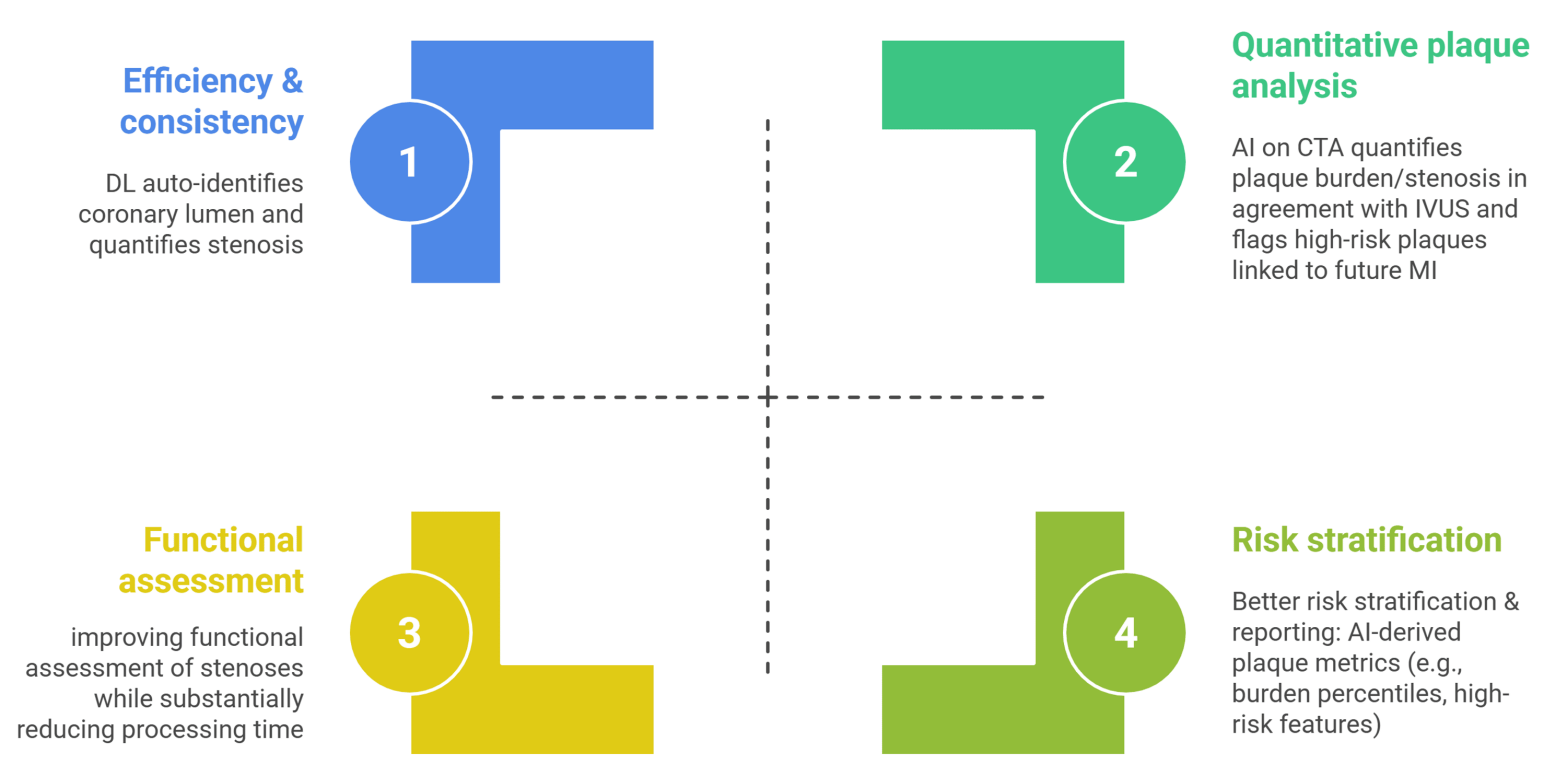
AI impact on coronary CTA AI: artificial intelligence; DL: deep learning; CTA: coronary computed tomography angiography; IVUS: intravascular ultrasound; MI: myocardial infarction; CT: computed tomography Image credit: The image was created by the authors Ahmed Khalifa and Mostafa Abdulaziz, summarizing the impact of AI on coronary CT angiography Source: [37-43]

AI-powered CCTA enhances prognostication by quantifying high-risk plaque features and burden. Studies show AI-derived plaque burden percentiles correlate with incident MI and predict long-term outcomes, outperforming conventional markers. A 2024 review confirmed that AI-assisted CTA improves diagnostic accuracy and risk stratification. Vendors are integrating these tools for structured plaque reports, with professional guidance emphasizing rigorous validation for clinical use (Figure 2) [40-43].

AI in Cardiac MRI and Perfusion Imaging

AI is advancing cardiac MRI for both infarct characterization (LGE) and ischemia assessment (stress perfusion) by automating segmentation and interpretation. DL models trained on labeled perfusion studies can detect inducible hypoperfusion at the segment level, achieve expert-level accuracy, and provide more reproducible quantification of ischemic burden. Inline DL pipelines now perform real-time myocardium segmentation and generate perfusion-defect maps during acquisition, streamlining workflow and enabling consistent, objective ischemia reporting [44].

Another notable work is by Singh et al., who created an “explainable” DL model for SPECT myocardial perfusion imaging that directly predicts patient outcomes (death or MI) instead of just detecting ischemia. The model was trained to predict the five-year risk of major adverse cardiac events (MACE), which included cardiac death and MI [36].

AI analyzes LGE MRI to quantify scarred myocardium from past infarcts, standardizing infarct size and transmurality assessment through DL segmentation. Combining LGE findings with other data via AI may better predict functional recovery after revascularization. For instance, ML integrates LGE features with clinical variables to determine if hibernating myocardium will improve after bypass surgery. AI can reduce manual contouring and detect subtle enhancements in general, even though ischemia-focused examples are less common [45-48].

AI in wearables and remote monitoring

Smartwatches with ECG capability and wearable patches can capture cardiac rhythm data outside the clinic. While most current FDA-cleared algorithms for wearables focus on arrhythmia (like atrial fibrillation detection), research is extending into ischemia detection. One approach uses AI to monitor subtle ST-segment changes or deviations in heart rate variability that might indicate myocardial ischemia. For instance, an experimental device called “miniECG,” a pocket-sized four-lead gadget, was tested with an AI model and showed promise in detecting acute coronary occlusion by simple placement on the chest [49].

Additionally, wearable chest patches and other ambulatory multilead ECG platforms have been paired with machine-learning models to detect transient ST-segment depression (silent ischemia) during daily activities, with early studies showing promising but imperfect performance on long-term ambulatory datasets [50-52]. Motion artifacts remain a major challenge for daily-life recordings and are an active area of methods and materials research [53,54]. Hardware feasibility includes soft, stretchable multilead chest patches and approaches that reconstruct diagnostic 12-lead ECG from wearable lead sets [8,9].

Hardware feasibility includes soft, stretchable multilead chest patches and approaches that reconstruct a diagnostic 12-lead ECG from wearable lead sets [55]. A parallel engineering trend is on-device (edge-AI) inference: energy-efficient microcontroller/ASIC designs have demonstrated real-time MI detection directly on the wearable, reducing reliance on cloud computation [56,57].

Another angle is using smartphone apps where patients input symptoms (like episodic chest pain) and wearables supply vital data, with AI algorithms determining the likelihood of an ACS. In low-risk chest pain populations, such an approach might rule out MI and reduce unnecessary ER visits. There are ongoing trials of AI-based chest pain triage tools on smartphones [30,31,58,59].

In a cohort of 1,066 participants, use of a mobile app that combined wearable data, machine-learning analytics, and continuous glucose monitoring was linked to better metabolic profiles, including improved glycemic levels, reduced variability, and fewer glycemic events [60].

Current limitations and future directions

A crucial question is whether using AI actually improves patient outcomes (e.g., fewer missed MIs, more appropriate catheterizations, reduced mortality). To date, most studies have focused on diagnostic performance rather than outcomes. Nonetheless, some early evidence is encouraging. By catching occlusion MIs that would have been missed, AI-ECG tools could reduce door-to-balloon times for those patients, theoretically improving survival [22].

Integrating AI is not without challenges. Workflow disruptions, alert fatigue, and over-reliance (or under-reliance) on AI are all possible. A known issue is that if the AI is treated as infallible, clinicians might overlook obvious clinical signs if the AI says “normal.” Conversely, if AI outputs are buried or hard to access, they will be ignored. Therefore, careful user-centered design is crucial; for example, one recommendation is to have AI generate a report that becomes part of the patient record (just like a lab result), rather than a separate app clinicians must open. Additionally, ensuring interoperability (AI working with different ECG machines, different imaging scanners, and EHR systems) is key to seamless integration [61-64].

AI models are only as good as the data they are trained on. In cardiology, large datasets often come from specific geographies or hospital systems and may not represent all patient groups. This can introduce bias. A 2024 perspective in npj Cardiovascular Health noted that biased algorithms can amplify healthcare inequities, performing worse in historically marginalized groups based on race, sex, or socioeconomic status [18].

Sources of bias include underrepresentation of certain groups in training data, or systemic biases in how care is delivered (for example, if minority patients have more unrecognized MI, an AI learning from outcomes might wrongly assume those patients have a lower risk because their events were missed clinically.

Emerging Innovations and Future Directions

The future of AI in cardiac ischemia detection promises revolutionary advances in multimodal diagnostics: algorithms are being developed to integrate EHRs, ECG, advanced imaging, wearable sensors, and circulating biomarkers into comprehensive risk models. Recent work reports AI-driven tools with superior diagnostic accuracy over traditional methods, while federated learning and explainable AI are highlighted as enablers of robust, scalable, and equitable deployment. In parallel, advanced algorithms are enhancing image acquisition, reconstruction, and interpretation across modalities and informing AI-driven decision-support systems that help clinicians craft personalized treatment plans from integrated imaging data [65-67].

Personalized medicine and continuous monitoring represent the next frontier in AI applications for cardiac ischemia management. The future lies in treatment strategies tailored to individual patient genetic, molecular, and imaging profiles, with multimodality imaging integrated with genomic and proteomic data providing a holistic view of the disease. Advances in wearable technology and remote monitoring devices will enable continuous assessment of cardiac function and early detection of ischemic events, with AI algorithms providing real-time alerts and actionable insights to improve patient management and outcomes. AI-enhanced tools are being developed for superior risk prediction by integrating multimodal data from clinical sources, patient-generated inputs, and biomarkers, offering more precise risk assessment and personalized care [66,67].

## Conclusions

The digital revolution in cardiac ischemia represents a transformative advancement in cardiovascular medicine, with AI demonstrating exceptional capabilities in enhancing detection, diagnosis, and risk stratification. AI-enhanced electrocardiogram analysis achieves notable accuracies and successfully identifies subtle ischemic patterns, including occlusion MIs that conventional methods often miss. Advanced cardiac imaging applications, particularly in coronary CT angiography and cardiac MRI, have revolutionized clinical workflows through automated plaque quantification and AI-powered FFR computation, while wearable devices enable continuous monitoring and real-time risk assessment outside traditional healthcare settings.

Despite these remarkable advances, several critical implementation challenges require careful consideration, including data bias, algorithmic equity concerns, workflow integration difficulties, and the need for rigorous prospective validation studies to demonstrate real-world clinical benefits. The transition from research performance metrics to demonstrable patient outcomes necessitates comprehensive clinical validation and careful user-centered design to address issues such as alert fatigue and overreliance on automated systems.
